# Accuracy of glomerular filtration rate equations for chronic kidney disease patients at the G3a stage: a single-center cross-sectional study

**DOI:** 10.1186/s13104-017-2400-8

**Published:** 2017-02-24

**Authors:** Yanna Dou, Xiran Sun, Dong Liu, Li Zhang, Jing Xiao, Genyang Cheng, Dahai Yu, Zhanzheng Zhao

**Affiliations:** 1 The Nephrology Center of the First Affiliated Hospital of Zhengzhou University, Zhengzhou, China; 2 Zhengzhou University Institute of Nephrology, Zhengzhou, China; 3 Arthritis Research UK Primary Care Centre, Research Institute for Primary Care & Health Sciences, Keele University, Keele, ST5 5BG UK

**Keywords:** G3a stage, Glomerular filtration rate equations, Accuracy, Chronic kidney disease

## Abstract

**Background:**

Kidney disease improving global outcomes provided a new classification for chronic kidney disease (CKD) by subdividing the G3 stage into G3a and G3b stages based on glomerular filtration rate (GFR) in 2012. Currently, a few methods are used to evaluate GFR, including measured GFR (mGFR) and estimated GFR (eGFR). One of the mGFR was ^99m^Tc-DTPA scintigraphy method and eGFR using GFR equations were used clinically. Equations were modification of diet in renal disease (MDRD), chronic kidney disease epidemiology collaboration (CKD-EPI), and Chinese adapted MDRD (C-MDRD). This study assessed the accuracy of three different equations for estimated glomerular filtration rate (eGFR) with mGFR using DTPA scintigraphy method as the standard in a population of Chinese chronic kidney disease patients at the G3a stage.

**Results:**

One hundred and twenty-two patients (age 52.0 ± 15.6 years, 69 were male) were determined as CKD stage 3 based on mGFR. Patients were divided into G3a (47 patients) and G3b (75 patients) subgroups. Bias between eGFR for CKD-EPI and reference mGFR was 0.92 mL/min and 95% limits of agreement was −38.82 to 40.67 mL/min. Bias between eGFR for C-MDRD and mGFR was 3.76 and 95% limits of agreement was −39.32 to 46.85 mL/min. Bias between eGFR for MDRD and mGFR was 3.53 and 95% limits of agreement was −43.35 to 50.4 mL/min. The CKD-EPI equation showed better diagnostic value with a greater area under the receiver operating characteristic curve (AUC: 0.763). AUC for MDRD and C-MDRD were 0.75 and 0.757, respectively.

**Conclusions:**

There were no obvious advantages in accuracy, sensitivity, and specificity for the diagnosis of patients at the G3a stage using the CKD-EPI equation.

**Electronic supplementary material:**

The online version of this article (doi:10.1186/s13104-017-2400-8) contains supplementary material, which is available to authorized users.

## Background

In 2012, kidney disease improving global outcomes provided a new classification for chronic kidney disease (CKD) by subdividing the G3 stage into G3a and G3b stages based on glomerular filtration rate (GFR). GFR for G3a is between 45 and 59 mL/min and GFR for G3b is between 30 and 44 mL/min. The risk for end-stage renal disease and death increased for patients at G3b more so than for patients at G3a. Go et al. reported the adjusted hazard ratio for death was 1.2 for patients at G3a (95% confidence interval (CI) 1.1–1.2) and 1.8 at G3b (95% CI 1.7–1.9) [1]. Over a median follow-up of 2.98 years, rates per 100 patient-years for end-stage renal disease were lower for G3a than G3b, at 0.6 (95% CI 0.1–1.0) vs. 1.4 (95% CI 0.8–2.1). Death prior to end-stage renal disease for G3a was 2.2 (95% CI 1.2–3.1; p < 0.001), which is lower than G3b, at 4.4 (95% CI 3.3–5.6; p < 0.001) [2]. Following from these classifications, it is important that patients are accurately assessed for G3 staging.

Currently, a few methods are used to evaluate GFR, including measured GFR (mGFR) and estimated GFR (eGFR). mGFR is determined by analysis of the clearance of some exogenous markers, such as inulin, or of alternative exogenous markers, such as iothalamate, EDTA, diethylene triamine pentaacetic acid (DTPA), and iohexol. These methods are, however, invasive, inconvenient, and expensive, and are unsuitable for use in routine clinical practice [3]. The most commonly used creatinine-based formulas include modification of diet in renal disease (MDRD) [4], chronic kidney disease epidemiology collaboration (CKD-EPI) [5], and the Chinese adapted MDRD equation (C-MDRD), the latter of which was validated as being more accurate than other MDRD equations for people of Chinese origin [6].

In this study, we compared the accuracy of eGFR using three equations and mGFR as the standard in Chinese CKD patients at the G3a stage.

## Methods

### Study design

This study was conducted at The First Affiliated Hospital of Zhengzhou University between June 2013 and July 2014. It was a cross-sectional study performed at a single center. mGFR based on ^99m^Tc-DTPA scintigraphy and eGFR using the GFR equations detailed below were assessed. Bias, sensitivity, and specificity of eGFR were analyzed with mGFR in participants at the G3a stage. The study was approved by the Review Board of The First Affiliated Hospital of Zhengzhou University and all participants provided informed consent in written format.

### Population

Chinese adult inpatients with CKD between the ages of 18 and 80 years were included. Patients who were not at the G3 stage based on mGFR were excluded. CKD and its stages were defined according to K/DOQI guidelines: (1) renal damage >3 months, established by structural or functional damage, with or without decrease in GFR, shown by histological anomalies and renal damage markers, including those found in blood, urine, or images; or (2) GFR <60 mL/min/1.73 m^2^ >3 months, with or without renal damage [7]. CKD was divided into five stages, including G3a and G3b stages.

### Studies

Fasting serum creatinine, ^99m^Tc-DTPA scintigraphy, and renal sonogram studies were performed. Blood samples and gammagraphic studies were performed at The First Affiliated Hospital of Zhengzhou University by the same professionals.

Dynamic gammagraphy with ^99m^Tc-DTPA is regarded as the gold standard [8]. ^99m^Tc-DTPA dynamic images were acquired using a detector of gamma camera with the patient in the supine position. Patients were injected with 200 μCi/kg (at least 2 mCi) of ^99m^Tc-DTPA and dynamic images were recorded in a 128 × 128 matrix format every second for 1 min and every 30 s for 20 min. Relative renal function was measured in a composite image (1–3 min after injection).

GFR was estimated by serum creatinine using the CKD-EPI equation [5], the MDRD [4] equation, and the C-MDRD equation [6]. Creatinine was determined using the dry chemistry sarcosine oxidase method with traceable calibration using a Roche Cobas 8000. The normal serum creatinine level is 20–115 µmol/L.

### Equations

1 $$\begin{aligned} &{\text{CKD-MDRD}}{:} \, {\text{ eGFR}}\,\left[ {{\text{mL}}\,{ \hbox{min} }^{ - 1} \,( 1.73 \,{\text{m}}^{ 2} )^{ - 1} } \right] \\ &\quad = 186 \times \left[ {{\text{Scr }}\left( {{\text{mg}}/{\text{dL}}} \right)} \right]^{ - 1.154} \times {\text{age}}^{ - 0.203} \times 0.742 { }\, \left( {\text{if female}} \right) \end{aligned}$$

2 $$\begin{aligned} &{\text{CKD-C-MDRD}}{:}\,{\text{eGFR}}\,\left[ {{\text{mL}}\,{ \hbox{min} }^{ - 1} \,( 1.73 \,{\text{m}}^{ 2} )^{ - 1} } \right] \\ &\quad = 175 \times \left[ {{\text{Scr }}\left( {{\text{mg}}/{\text{dL}}} \right)} \right]^{ - 1.234} \times {\text{age}}^{ - 0.179} \times 0.79\,\left( {\text{if female}} \right) \end{aligned}$$

3 $$\begin{aligned} &{\text{CKD-EPI}}:\,{\text{eGFR}}\,\left[ {{\text{mL}}\,{ \hbox{min} }^{ - 1} \,( 1.73 \,{\text{m}}^{ 2} )^{ - 1} } \right] \\ & \quad= \, 141 \times { \hbox{min} }\,\left( {{\text{Scr}}/\kappa , 1} \right)^{\alpha } \times { \hbox{max} }\,\left( {{\text{Scr}}/\kappa , 1} \right)^{ - 1.209} \\ \quad \times 0.993^{\text{age}} \times 1.018\,\left( {\text{if female}} \right)\_ 1.159\,\left( {\text{if black}} \right) \\ \end{aligned}$$

(Female: κ = 0.7, α = − 0.329; male: κ = 0.9, α = − 0.411; min indicates the minimum Scr/κ or 1, and max indicates the maximum Scr/κ or 1). Scr: serum creatinine.

### Statistical analysis

Continuous data are expressed as mean ± standard deviation or medians (25, 75%). The independent *t* test and nonparametric tests were used to compare general information between the G3a and G3b groups. Bland–Altman plots were used to compare different estimates of GFR (bias and 95% limits of agreement). Results were considered significant if p ≤ 0.05. Accuracy was calculated as the proportion of eGFR within 15, 30, and 50% of measured GFR (mGFR) (P15, P30, and P50, respectively). The McNemar test was used to compare P15, P30 and P50 values of eGFR against P15, P30 and P50 values of mGFR. A receiver operating characteristic (ROC) curve was plotted and the area under the ROC curve (AUC) was calculated.

## Results

Four hundred and sixty-three CKD patients were screened. Based on results of mGFR, 122 patients (age 52.0 ± 15.6 years, 69 were male) were determined as CKD stage 3 (Additional file 1). These patients were divided into either G3a (47 patients) or G3b stages (75 patients). The general characteristics of the two groups are shown in Table 1. There were no significant differences in age, height, body surface area, or weight between groups. eGFR values for G3b were lower than that for G3a.

**Table 1:** General characteristics of CKD patients at the G3 stage

	G3b (30–44 mL/min)	G3a (45–59 mL/min)	p value
n	75	47	
Age (years)	53.8 ± 15.4	49.2 ± 15.7	>0.05
Height (cm)	165.8 ± 7.0	165.7 ± 7.6	>0.05
Weight (kg)	65.9 ± 12.0	66.5 ± 10.2	>0.05
Body surface area (m^2^)	1.7 ± 0.2	1.7 ± 0.1	>0.05
GFR-DTPA (mL/min)	38.0 ± 4.3	52.0 ± 4.7	<0.05
GFR-EPI (mL/min)	30.0 (12.0, 40.9)	55.4 (37.2, 74.1)	<0.05
GFR-MDRD (mL/min)	23.7 (10.2, 35.7)	44.3 (27.6, 61.9)	<0.05
GFR-C-MDRD (mL/min)	29.1 (12.5, 43.9)	54.5 (33.9, 76.2)	<0.05

Data was expressed as mean ± SD or medians (25, 75%)

Bias between eGFR_CKD-EPI and mGFR was 0.92 mL/min and 95% limits of agreement was −38.82 to 40.67 mL/min. Bias between eGFR_C-MDRD and mGFR was 3.76 and 95% limits of agreement was −39.32 to 46.85 mL/min. Bias between eGFR_MDRD and mGFR was 3.53 and 95% limits of agreement was −43.35 to 50.4 mL/min.

P15 values at the G3a stage were 38.3, 38.3, and 42.6% for CKD-EPI (Eq. 3), CKD-C-MDRD (Eq. 2), and CKD-MDRD (Eq. 1), respectively (all p > 0.05) (Table 2). P30 values at the G3a stage were 40.4, 42.6, and 44.7% for CKD-EPI (Eq. 3), CKD-C-MDRD (Eq. 2), and CKD-MDRD (Eq. 1), respectively (all p > 0.05) (Table 2). P50 values at the G3a stage were 55.4, 48.9, and 48.9% for CKD-EPI (Eq. 3), CKD-C-MDRD (Eq. 2), and CKD-MDRD (Eq. 1), respectively (all p > 0.05) (Table 2).

**Table 2:** Comparison of the accuracy of different GFR equations

	15%			30%			50%		
	G3	G3a	G3b	G3	G3a	G3b	G3	G3a	G3b
CKD-EPI	41.8	38.3	54.7	50	40.4	60	56.6	55.4	62.7
CKD-C-MDRD	44.3	38.3	56	51.6	42.6	60	55.7	48.9	65.3
CKD-MDRD	44.3	42.6	57.3	51.6	44.7	58.7	55.7	48.9	65.3
p value	>0.05	>0.05	>0.05	>0.05	>0.05	>0.05	>0.05	>0.05	>0.05

**Fig. 1:**
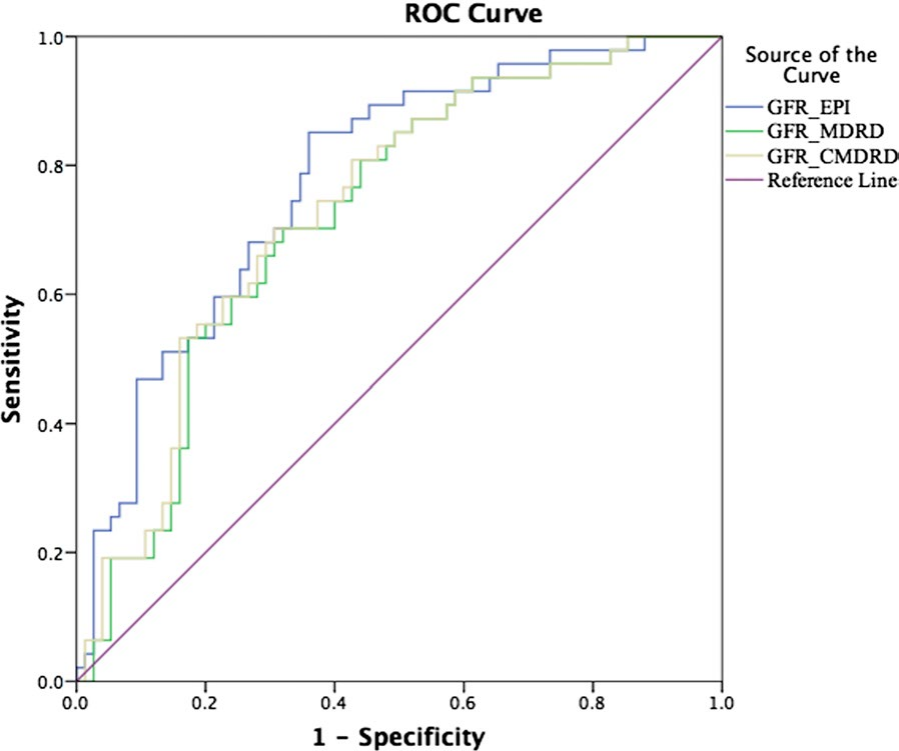
ROC curve for different eGFR values

The CKD-EPI equation (Eq. 3) showed better diagnostic value, having a greater AUC (AUC: 0.763). The AUC for CKD-MDRD and CKD-C-MDRD were 0.75 and 0.757, respectively (Fig. 1).

## Discussion

Our study found that the bias between eGFR_CKD-EPI and mGFR was smaller than the other equations examined; however, there were no statistical differences in sensitivity, specificity, and ROC curve among the three equations for G3a.

The results of the current study are consistent with previous studies that found no significant differences in CKD stage 3 using different GFR equations. Levey et al. reported that the CKD-EPI equation was as accurate as the MDRD Study equation in a subgroup with an eGFR of less than 60 mL/min/1.73 m^2^. There was no difference between the CKD-EPI and MDRD Study equations in ROC curves to detect GFR <60, <45, <30, and <15 mL/min/1.73 m^2^. AUC were 0.96, 0.97, 0.97, and 0.98, respectively, for both equations [5]. Ma et al. found no significant differences in the percentages of misclassifications between the C-MDRD and MDRD equations when assessing CKD stages 3–5 [6].

One possible reason for the findings may be because of the distribution of the CKD stage in the reference population. The distribution of CKD stage or mGFR of the enrolled patients had an influence on the resulting equation. However, in the development dataset of the C-MDRD, MDRD, and CKD-EPI equations, mGFR was evenly distributed in CKD stage 3. In the study by Levey et al., the percentage of reference population in CKD stage 3 was about 33.2% [5] and in the study by Ma et al. the percentage was about 28.8% [6].

Creatinine value is another factor that may impact the accuracy of eGFR. In assessment of the three equations, creatinine values were either measured using the Roche enzymatic method or calibrated to the Roche enzymatic method. For the MDRD equation, serum creatinine was measured using a kinetic alkaline picrate assay [9] and the researchers re-expressed the four-variable (modified) MDRD Study equation that we used in the current research with the Roche enzymatic method [10]. For the C-MDRD equation, creatinine levels were measured at a single laboratory using a Hitachi 7600 analyzer and Jaffe’s kinetic method. The researchers randomly selected 57 fresh-frozen plasma samples from their specimens and analyzed them at both their own laboratory and the Cleveland Clinic Research Laboratory (Cleveland, OH, USA) [6]. The creatinine value at their laboratory was calibrated to the creatinine value measured by the Cleveland Clinic Research Laboratory where the MDRD Study samples were measured. For the CKD-EPI equation, for all studies, serum creatinine was recalibrated to the standardized creatinine measurement using the Roche enzymatic method (Roche-Hitachi P-Module instrument with Roche Creatininase Plus assay, Hoffman-La Roche, Ltd., Basel, Switzerland) at the Cleveland Clinic Research Laboratory. For the current study, we used the Roche enzymatic method at our laboratory. Based on this, the discrepancy of eGFR caused by the creatinine value is less. However, the creatinine generation is associated with the muscle mass [11], and the influence of muscle mass on creatinine estimation cannot be completely ignored.

The biases between mGFR and eGFR using the C-MDRD and MDRD equations are greater than that using the CKD-EPI equation. One possible reason may be mGFR data obtained using different methods, which may influence the bias of the different eGFR equations. The method used to obtain mGFR should be taken into consideration when evaluating GFR estimating equations. The MDRD study used renal clearance of ^125^I-iothalamate, the Chinese study used plasma clearance of ^99m^Tc-DTPA, and the CKD-EPI group used iothalamate in the development of datasets. Chen et al. and Shanshan Dai et al. confirmed that GFR measured by ^99m^Tc-DTPA plasma clearance and ^99m^Tc-DTPA dynamic imaging significantly overestimates GFR when compared with inulin clearance, especially in groups with low GFR, low body mass index, and a younger age [12, 13]. Soveri et al. suggested that renal clearance of DTPA, renal clearance of iohexol, and plasma clearance of inulin had sufficient accuracy (limited evidence) [14]. The muscle mass may also contributed to the accuracy of CKD-EPI equation [5]. Because the average muscle mass among healthy persons in the cohort of CKD-EPI equation was higher than other equations, and the CKD-EPI equation includes the indicators of age, race and sex and these variables are associated with muscle mass.

There are some limitations of this study. First, this is a single center study, and a multi-center study is necessary to validate the results. Second, though the CKD-EPI equation showed less bias in the G3a stage, but this equation could not avoid the disadvantage of serum creatinine, that the value of serum creatinine would be affected by the muscle mass. And Segarra et al. suggest that the use of equations based on cystatin C (CysC) is more appropriate in hospitalized patients to estimate GFR, since these equations are much less dependent on patient’s nutritional status or muscle mass than the CKD-EPI equation [15]. We will explore the accuracy of eGFR equations based on CysC in the G3a stage.

## Conclusions

The bias between mGFR and eGFR using the CKD-EPI equation at the G3a stage was less than that for the other two equations. However, there was no obvious advantage in accuracy, sensitivity, and specificity for diagnosis at the G3a stage using the CKD-EPI equation.

## Additional file

**Additional file 1.** The initial data of the GFR evaluation in G3A stage. The data is all the initial information of GFR evaluation in G3A stage of Chinese patients. It includes the general information of the patients, and the measuring variables of the patients.

## Abbreviations

CKD: chronic kidney disease
GFR: glomerular filtration rate
MDRD: modification of diet in renal disease
CKD-EPI: chronic kidney disease epidemiology collaboration
C-MDRD: Chinese adapted MDRD equation
DTPA: diethylene triamine pentaacetic acid
mGFR: measured GFR
eGFR: estimated GFR
ROC: receiver operating characteristic
AUC: area under ROC curve
